# Benchmarking for Strain Evaluation in CFRP Laminates Using Computer Vision: Machine Learning versus Deep Learning

**DOI:** 10.3390/ma15186310

**Published:** 2022-09-11

**Authors:** Jónatas Valença, Habibu Mukhandi, André G. Araújo, Micael S. Couceiro, Eduardo Júlio

**Affiliations:** 1 CERIS, IST-ID, University of Lisbon, 1049-003 Lisboa, Portugal; 2 Institute of Systems and Robotics, University of Coimbra, 3030-290 Coimbra, Portugal; 3 Ingeniarius, Lda, 4445-147 Porto, Portugal; 4 CERIS, IST, University of Lisbon, 1049-001 Lisboa, Portugal

**Keywords:** machine learning, deep learning, computer vision, CFRP laminates, strengthening RC, strain monitoring

## Abstract

The strengthening of concrete structures with laminates of Carbon-Fiber-Reinforced Polymers (CFRP) is a widely adopted technique. retained The application is more effective if pre-stressed CFRP laminates are adopted. The measurement of the strain level during the pre-stress application usually involves laborious and time-consuming applications of instrumentation. Thus, the development of expedited approaches to accurately measure the pre-stressed application in the laminates represents an important contribution to the field. This paper proposes and benchmarks contact-free architecture for measuring the strain level of CFRP laminate based on computer vision. The main objective is to provide a solution that might be economically feasible, automated, easy to use, and accurate. The architecture is fed by digitally deformed synthetic images, generated based on a low-resolution camera. The adopted methods range from traditional machine learning to deep learning. Furthermore, dropout and cross-validation methods for quantifying traditional machine learning algorithms and neural networks are used to efficiently provide uncertainty estimates. ResNet34 deep learning architecture provided the most accurate results, reaching a root mean square error (RMSE) of 0.057‰ for strain prediction. Finally, it is important to highlight that the architecture presented is contact-free, automatic, cost-effective, and measures directly on the laminate surfaces, which allows them to be widely used in the application of pre-stressed laminates.

## 1. Introduction

**Figure 1:**
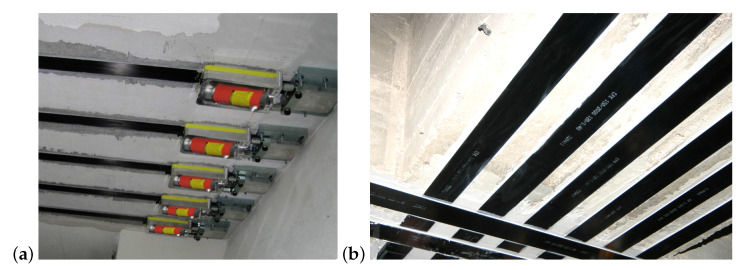
Application of Carbon-Fiber-Reinforced Polymer (CFRP) laminates: (**a**) pre-stress application on the laminates; (**b**) final application of the laminates (images provided by S&P, Clever Reinforcement Iberica—Materiais de Construçao, Lda.).

The use of Fiber-Reinforced Polymer (FRP) composites is widely used in the reinforcement of reinforced concrete (RC) structures, and can provide a different solution in terms of modulus of elasticity, tensile strength, and bond behavior. For example, FRP rods have been successfully applied to replace steel rods in several fields and industries because of their high performance in terms of durability, and the problems they avoid related to the corrosion of steel [1]. The use of FRP composites also presents good results for cracking and deformation behavior [2]. The main advantages are related to its light weight, high strength, corrosion resistance, creep resistance, and fatigue resistance, especially the Carbon-Fiber-Reinforced Polymer (CFRP) [3]. The Advanced Finite Element Method (FEM) based on artificial neural networks (ANNs) are also used to predict the strength and progressive damage behavior of CFRP laminates [4]. The particular case of pre-stressed CFRP laminates is a common solution for strengthening large-span RC structures (Figure 1). Pre-stress is applied through hydraulic jacks with pressure control provided by a manometer. In the most relevant cases, traditional instrumentation, such as transducers and strain gauges, is used to directly control the applied strain. However, it is a time-consuming and laborious solution and, in the majority of cases, the pre-stress is indirectly estimated by the pressure applied. The bibliography proposes the use of fiber-optic sensors as an effective tool to monitor strain [5]. However, the precision of monitoring decreases over time as the fiber-optic degrades, and it is an expensive solution. Other options focus on measuring the electrical resistance condition of CFRP material or its potential difference [6]. These methods have been also suggested for monitoring the durability of CFRP by analyzing their electrical resistance change over time. The advantages of self-sensing systems can be obtained at a low cost, since the carbon fibers are sensors and reach a high level of precision, and also because the strains are directly measured in the fibers. Nevertheless, in line with other sensing methods, this implies that an elaborate set-up is necessitated, since this method requires the installation of electrodes in CFRP. Thus, contact-free solutions, such as image-based methods to assess the pre-stress application in CFRP laminates, are attractive.

Structural engineering has applied image-based methods, mainly image processing and photogrammetry, for monitoring displacements, strains, and cracking [7,8]. Despite these developments, the traditional instrumentation is still widely applied. In recent years, machine learning, deep learning, and convolutional neural networks (CNN) have been applied for structural health monitoring, a trend already verified in other research areas [9,10,11,12,13]. For example, computer vision and machine learning were applied for the post-disaster analysis of a building, allowing for fast diagnosis [14]. In general, the procedures presented overcome the problems commonly related to image processing and often achieve high accuracy rates, above 95%. One of the major problems reported is the high dependence of the dataset for training, due to its difficulty to obtain on a large scale, as well as labeled, i.e., datasets with ground truth associated. Thus, to evaluate and benchmark architectures based on both machine learning and deep learning algorithms represent a relevant contribution for strain evaluation in CFRP laminates.

In this paper, a contact-free architecture for measuring the strain applied in CFRP laminate is proposed based on the images captured by a low-resolution camera. The goal is to develop an automatic and cost-effective solution that can deliver accuracy with high confidence strain values during a pre-stress application on the laminates. The architecture proposed was developed around computer vision algorithms and was tested on a dataset of synthetic images of the laminates. The dataset resolution and introduced noise were defined to mimic real-world specifications. The architecture is evaluated by benchmarking several state-of-the-art and well-known artificial intelligence algorithms, from machine learning to deep learning approaches.

The paper is divided as follows: after the introduction, the next section leads with the definition and conceptual distinction between machine learning and deep learning. The following section describes creating the dataset process, which consists of synthetic images generated to mimic the real images captured by the low-resolution camera. Then, Section 4 presents the implemented solutions—both machine learning algorithms for computer vision and deep learning. Afterwards, the results of the implemented solutions and the algorithms are benchmarked. Final remarks, findings, and comments on the successes of the research and objectives are proposed in Section 6.

## 2. Conceptual Distinction of Traditional Machine Learning and Deep Learning

Today, artificial intelligence (AI) is one of the most active and attractive research domains. In the earlier times of artificial intelligence, the main focus was to solve problems that can be mathematically formalized, i.e., problems that are intellectually difficult for human beings but straightforward for computers. Nevertheless, the real challenge lies in solving the problems that are easy to solve intuitively for human beings, such as recognizing faces in images or spoken words. To understand of the fundamentals of this field, it is important to distinguish the conceptual distinction of traditional machine learning and deep learning. Artificial intelligence refers to a study of an intelligent device that mimics human behavior, perceives its environment, and has the decision-making ability to take actions to solve complex problems [15]. AI studies may include reasoning, planning, natural language processing, learning, perception, and many others. The commonly used approaches are based on mathematical optimization, probability, and statistical methods. AI research is a multidisciplinary field that includes computer science, mathematics, psychology, linguistics, philosophy, neuroscience, artificial psychology, and many others. Early AI studies focused on hard-coded statements in formal languages which rely on logical inference rules, known as the knowledge-based approach [16].

**Figure 2:**
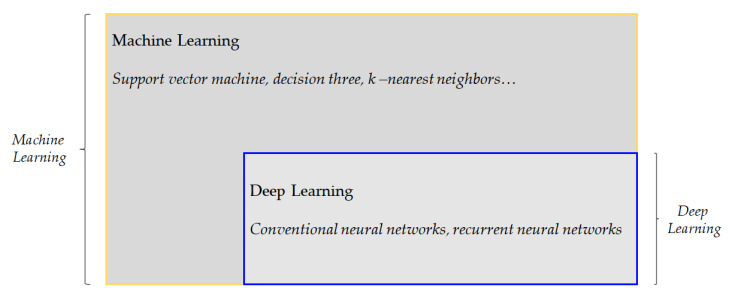
Machine learning vs. deep learning algorithms.

However, there has been a limitation in this approach—for humans, it is complex to formalize and explicate the knowledge associated with intuitively easy tasks [17]. Machine learning (ML) overcomes this limitation and relieves humans from this task. The machine learning term refers to the “ability to learn without being explicitly programmed” [18]. In more detail, ML refers to a computer that improves its performance by learning from provided problem-specific training data, in that, it aims to automatically create model-building to perform cognitive tasks by finding hidden insights and patterns without being explicitly programmed. Machine learning can be distinguished into three types: supervised learning, unsupervised learning, and reinforcement learning:

- Supervised learning a training dataset that includes an output with labeled answers or target values for the input data. The dataset consists of pairs of input–output data and are used in the training to build the ML model. Afterwards, the target variable or the class will be predicted based on this ML model in different types of problems, such as regression or classification.
- Unsupervised learning is supposed to predict the output without any existing specification or supervision. Thus, in this technique, the correct answer is not given to the system. The system detects the patterns and structural information that share common properties.
- Reinforcement learning instead of providing a training set, the system describes the algorithm with three components: a goal, a list of allowed actions, and the environmental constraints. With these specifications, the ML model experiences the process of achieving goals based on trial and error. The ML model objective is to choose actions that maximize the expected reward and learn the best policy. The notable progress in computer hardware technologies and explosive increment in available data had required slightly more advanced algorithms in ML, such as deep learning, which outperforms its predecessors [19]. The terms machine learning and deep learning have a historical and methodological hierarchical relationship, which is presented in Figure 2 [16].

**Figure 3:**
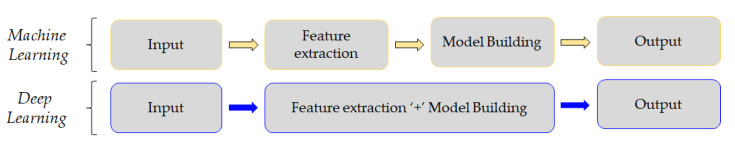
Model building process in machine learning and deep learning.

In traditional machine learning, the system success highly depends on the representation of a good dataset. This implies that feature engineering plays a key role in traditional machine learning, which focuses on building specific features from raw data with human effort. In comparison with traditional machine learning, as shown in Figure 3, deep learning algorithms automatically perform feature extraction, implying minimal effort and knowledge from humans to extract the required distinct features [20]. Deep learning algorithms mimic the human brain. The brain has a layered architecture that consists of mathematical representations of connected processing units called artificial neurons. These connections between neurons are similar to synapses in the brain, whose signal strength can be adjusted during the learning process. This nested neural network architecture has an input layer, an output layer, and hidden layers. The number of neurons and layers are the model’s hyperparameters, which must be set manually or determined in an optimizing way [21]. In deep learning, besides it having more than one hidden layer, it also has advanced characteristics, such as using advanced neurons and using multiple activation functions that enable the algorithm to extract features automatically. Therefore, even though traditional machine learning provides good results, especially in the case of limited low-dimensional training data, deep learning is commonly preferred in the case of large and high-dimensional data, such as images, video, speech, text, and audio [22].

## 3. Dataset

Acquiring high-quality data with the required volume and the corresponding ground truth for learning tasks is challenging. Thus, generating meaningful synthetic images for training networks quickly and containing ground truth annotations throughout the whole dataset are solutions for evaluating the algorithm’s performance. For a proper evaluation, those data must simulate possible real-world scenarios, such as camera positions, environments, and actions applied.

### 3.1. Image Settings

**Figure 4:**
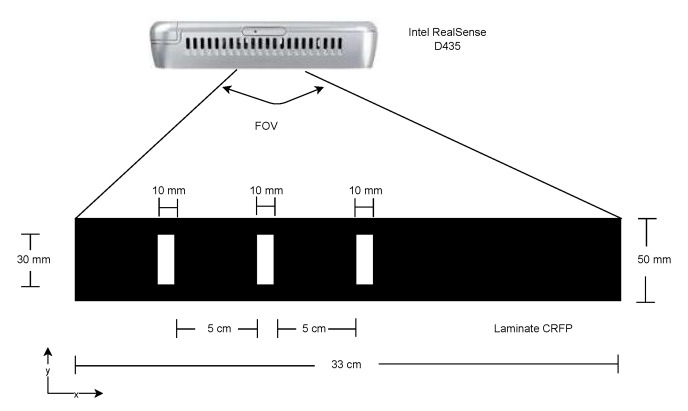
Set up for image acquisition.

A set of synthetic images was generated with a single requirement to simulate the acquisition with a RealSense D435 Camera that will be used in real cases after selecting the best architecture. Thus, the images should recreate an acquisition with a sensor size of 1751 px × 1493 px, and a focal length of 1.93 mm (Figure 4). Further, it was considered that the camera was placed 150 mm above the laminate. This results in a field-of-view (FOV) that was 330 mm wide and had a spatial resolution of 4 px/mm. We decided to generate synthetic images with a 1320 px × 200 px resolution and a printed pattern of three parallel stripes. The stripes, before stretching, had a 10 mm width and 30 mm length, and spaced 50 mm apart (Figure 4).

### 3.2. Digital Deformation and Noise

**Figure 5:**
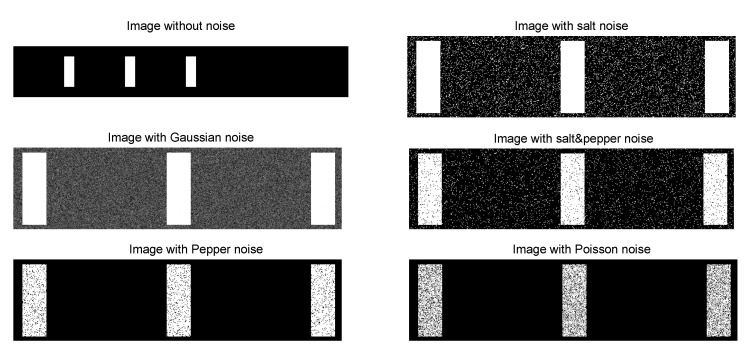
Synthetic images with different types of noises.

To simulate the strain induced in the laminates, the images produced were stretched in the direction of its major axis (*x*-axis), from 0‰ to 10‰, with increments of 0.1‰ applied in 330 mm of laminate length (in a total of 101 stages of deformation). To emulate that, the image was resized with a bi-cubic interpolation by multiplying each pixel position by a constant factor *dx* [23], i.e., 1 for a imposed strain of 0‰, 1.001 for 1‰, and so on. Further, different noise types were introduced to the synthetic images, aiming to mimic real-world conditions, e.g., lighting conditions, since images could be totally or partially exposed to light or shadows and thermal effects on the sensors, depending on the spectral resolution. The following noise types were applied: (i) Gaussian noise, to reproduce the effect of thermal noise due to camera sensor heating [24]; (ii) salt noise, to simulate images with random overexposed bright pixels [25]; (iii) pepper noise, to mimic underexposed dark pixels at random locations [25]; (iv) salt and pepper noise, that combines the last two [25]; (v) speckle noise, that reproduces the effect of the interference phenomenon—speckle—which occurs at wavelength scale due to the surface roughness [26]; and (vi) Poisson noise, which provides the statistical nature of electromagnetic waves (RealSense D435 camera capture infrared waves) [27] (see Figure 5).

### 3.3. Training, Validation, and Testing

The dataset was generated by creating one synthetic image without noise (i.e., original image) and 29 noisy images for each strain step, leading to 30 images for each imposed strain level (0‰ to 10‰). This results in a total of 3030 images—30 images for each of the 101 imposed strain levels. The algorithms adopted to benchmark rely on three subsets of the main dataset: training, validation, and testing. Therefore, from the original dataset comprising 3030 images, the original image and 19 noisy images for each strain step were used for training, while the remaining 10 noisy images, for each imposed strain level were used for testing. This leads to a total of 2020 images for the training dataset and 1010 images for the testing dataset. The training dataset, on the other hand, was further divided into 90% for training (1818 images), using the remaining 10% for validation (220 images). It is noteworthy that the validation set was only used after each training step of the models with the intent to evaluate which of them performs the best. The models go back to the training step to improve their result according to the performance assessed with the validation set. For reporting the results, the best-performing models are used for predictions on other unseen data; the final group of data is called the test set.

## 4. Methodology

### 4.1. Architecture

**Figure 6:**
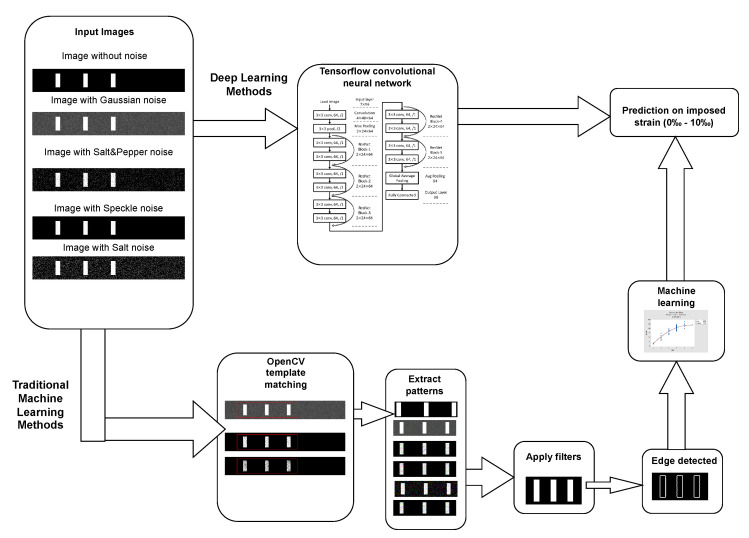
Illustration of the overall architecture.

The overall architecture proposed for measuring the strain at the CFRP laminates, benchmarking machine, and deep learning algorithms for computer vision are summarized in Figure 6. It starts from all images of the dataset for all strain levels considered. The strain can be estimated by using both traditional machine learning and deep learning. For traditional machine learning, the images require pre-processing procedures of template matching to extract one pattern among many, denoising the images, enabling feature extraction, and ending with the machine learning estimation of the strain. In the case of deep learning, after training, images were labeled as the input, and an estimation of the strain was directly performed.

### 4.2. Machine Learning with Regression Methods for Computer Vision

**Figure 7:**
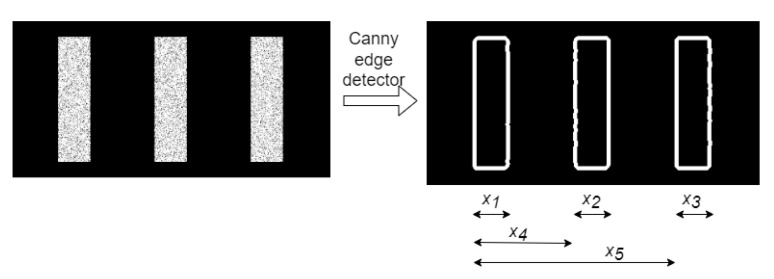
Image before and after applying Canny Edge Detector.

The shapes of the stripes in the images and its edges were extracted to compute five measurements variables, namely, the widths of the stripes and the distance between the first stripe and the second and third stripe (Figure 7). Then, the strain values were calculated by the average, or weighted average, of the five variables selected by obtaining their linear combination. This approach was used because, while stretching the laminate, the imposed strain should be linearly distributed along the *x*-axis of the laminate. However, this procedure fails to estimate the imposed strain correctly just by the linear combination of each variable. A suited weight approach was used to compute each variable; this led to a desirable root mean square error (RMSE) between the predicted and imposed strain. Thus, the input [*X*] comprises the set of variables handpicked by the users—the stripe’s width, position, distances between stripes, and the corresponding imposed strain are used as the target variables [*y*]. Machine learning with regression methods was adopted, because strain levels are continuous and not categorical to extracted variables; then, an optimization algorithm was applied by a non-linear function to provide the most adequate relation between variables and strain values. This is reached by computing the weights, leading to the minimum error between the predicted and the imposed strain. The architecture test, Polynomial Regression, Fully Connected Neural Network, Support Vector Regression (SVR), Decision Tree, and Random Forest were used in this optimization. In addition, due to the well-documented sensitivity of these methods to noise, images were filtered before extraction using a Canny Edge Detector.

#### 4.2.1. Filtering

Traditional machine learning is unable to detect the stripes due to the noise added to the images. In this case, several filters were required to remove the noise and enable the algorithms to perform. Two filters were used: (i) The Non-Local Mean Filter, which replaces the pixel color by the weighted average of colors of similar pixels [28], i.e., the weights computed by similar Gaussian functions. This filter allows most types of noises to be removed while preserving the edges. (ii) The Median Blur Filter, which replaces the central pixel of a kernel window by the median of all pixels; this was highly effective at removing salt-and-pepper noise [29].

#### 4.2.2. Edge Detection

The features for the machine learning methods were extracted using a Canny Edge Detector. The pattern, which was obtained with Template Matching [30] and a Canny Edge Detector [31], was applied to define the location and shape of each stripe. The imposition of the strain on the laminate resulted in changes in the distance between the stripes and changes in their widths. These geometric alterations reproduce the strain level of the laminate. The five variables mentioned are measured and used as input features to a traditional machine learning algorithm (see Figure 7).

#### 4.2.3. Machine Learning Algorithms

Five machine learning algorithms were tested to solve the regression problem, wherein the five input features extracted from the images here are represented as $x_{i}$, i=1,…,5. All the algorithms provide a target value [*y*], which represents the strain step related to a given set of input features. The next paragraphs summarize each of the five ML algorithms implemented.

Polynomial regression → consists of a linear regression algorithm with polynomial features that intends to achieve a linear relationship between input features [*x*] and the target variable [*y*] [32]. The polynomial features enables a non-linear function to be found to map the five features ($x_{i}$) and estimate the strain values ($y_{i}$) under a certain domain. Polynomial regression combines the features as $\theta_{0}+\theta_{1}nx_{1}^{n}+\theta_{2}nx_{2}^{n}+\theta_{3}nx_{3}^{n}+\theta_{4}nx_{4}^{n}+\theta_{5}nx_{5}^{n}+\theta_{6}x_{1}x_{2}+\theta_{7}x_{1}x_{2}x_{3}+\theta_{8}x_{1}x_{2}x_{3}x_{4}+\ldots =h\left(x\right)$, where n=1,…,4, *θ*s are model coefficients, and h(x) is the value predicted by the model.

Decision Tree Regression → The model is created from a supervised learning method to predict the value of a target variable by learning simple heuristics inferred from data [33,34]. The learned rules are used on splitting tree branches, minimizing the cost function by split the features and applying a recursive binary split. A minimal-cost-complexity pruning method was used to reduce the model variance The algorithm was able to learn a non-linear relationship between features and a target variable without parameters, e.g., between the five features extracted and the target variable. A single optimal hyperparameter was found and tuned, corresponding to the depth of the tree. This optimal maximum depth was obtained by cross-validation, and the optimal tree used for inference on the testing dataset.

Random Forest Regression → This consists of a meta estimator that fits decision tree classifiers, or regressions, on several subsamples of the dataset, using averaging to control variance and improve accuracy [35]. Since the training data can overfit, random forest averages the predictions from several trees to reduce the variance. The algorithm functions like Bootstrap Aggregation (Bagging) and each tree uses all the features in the dataset. A de-correlation was performed to randomly select a subset of features in each split of each tree, avoiding tree correlation. Similar to decision trees, hyperparemeter tuning was used to achieve the optimal number of trees and the optimal subset of features.

Support Vector Regression → This aims to locate the hyperplane, f(x) that better fits a maximum number of data points [36]. It works as a maximum margin algorithm, whereby the best fit correlates to the hyperplane closest to the majority of points—this is as smooth as possible and deviates maximally from the targets [$y_{i}$] by ε. The input points are transformed by a kernel and using the Support Vector Machines for computational efficiency:

(1) $$f\left(x\right)=w^{T}h\left(x\right)+b$$

where *h* is the function which maps the d-dimensional feature space, *w* represents a weight vector, and *b* is the bias term.

Fully Connected Neural Network (FCNN) → This is architecture created using hyperparameter tuning to compute an optimal number of neurons per layer, the number of hidden layers, the network learning rate (η), and optimization algorithm. The optimal FCNN was created by combining the already mentioned optimal hyperparameters to perform predictions on the testing dataset. The input was again the five features extracted from the strips pattern and the target values the strain values.

### 4.3. Deep Learning with Regression for Computer Vision

Contrary to traditional computer vision, deep learning is directly fed by images and not by a detail-oriented selection of features in the images [16,37]. Thus, deep-learning-supervised algorithms have been adopted, where images are used as input, [*X*], and their corresponding strain levels are the target variable [*y*]. The deep learning algorithm is responsible for automatically extracting the features through its convolution kernels, also known as weights, and estimating a function capable of relating these features, multiplied by the weights, to the strain levels. The architectures presented use two-dimensional convolutional neural networks (CNNs), dropout layers, maxpool layers, batch normalization layers, and a fully connected layer as building blocks. A brief description of the deep learning algorithms implemented is presented in the following paragraphs.

GoogLeNet with regression → GoogLeNet is a CNN architecture developed at Google which uses inception modules. In other words, it uses an architecture with different types of kernel size, such as 1 × 1, 3 × 3, 5 × 5, …, and a MaxPooling layer by concatenation in the same module [38]. Despite being very deep, GoogLeNet applies a reduced number of parameters (about 6 million parameters, making it computationally less expensive) compared with many other CNNs that are shallower due; this is due to its utilization of inception modules. GoogLeNet is very powerful, since inception layers include all types of kernel sizes, and the MaxPool layer is applied in a single inception module. In the work presented, a linear activation function with the mean square error loss replaces the final layer’s activation function to apply the GoogLeNet perform regression tasks. The parameters of the training using GoogleNet and ResNet are summarized in Table 1.

**Figure 8:**
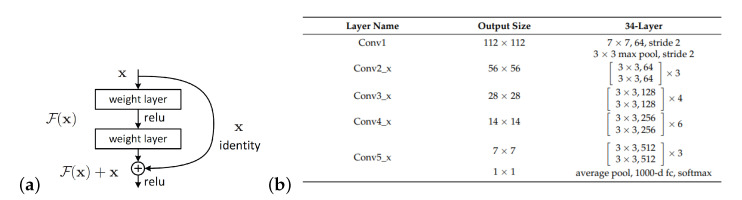
ResNet34: (**a**) “Basic-Block” building block; (**b**) The structure of ResNet34 (from [41]).

ResNet with Regression → ResNet very deep neural networks to be trained, considering more than a hundred layers without vanishing gradient problems [39]. These problems arise when the weights of the layers converge towards zero and the gradient flattens out [40]. ResNet solves the problem with a skip connection technique and, like for GoogLeNet with regression, the final layer’s activation function is replaced by a linear activation function with a mean square error loss and 34 layers—known as ResNet34 with regression). ResNet34 is composed of the residual building block and, to diminish the gradient disappearance problem caused by the depth increase in the neural networks, the residual buildiing block uses a shortcut connection to skip the convolutional layers. The benefit of this infrastructure is to provide CNN structures that are constructed with more flexibility. The basic block of ResNet34 is shown in Figure 8a, which comprises convolutional layers, batch normalizations (BNs), a rectified linear unit (ReLU) activation function, and a shortcut. Equation (1) indicates the output of the residual building block.

(2) y=f(x)+x

where *x* and *y* are the input and output of the residual function, respectively. The residual function represented as *f*, and the ResNet34 structure is presented in Figure 8b.

For the integration of deep learning algorithms, TensorFlow (https://www.tensorflow.org, accessed on 24 April 2022) and Scikit-Learn (https://scikit-learn.org, accessed on 24 April 2022) platforms were used. TensorFlow is an open source platform with a flexible and comprehensive ecosystem of tools and libraries, that allows ML-based applications to be built efficiently. Besides TensorFlow, Scikit-learn was also employed due to its interface, which makes it easy to deploy supervised and unsupervised algorithms in Python. It is also an open source library that focuses on modeling data.

## 5. Analysis of Results

### 5.1. Machine Learning

In the pre-processing step, a Canny Edge Detector was used in an attempt to remove noise with a Gaussian kernel. This filter is usually successful in removing Poisson noise and Speckle noise, but does not denoise pepper noise effectively. Furthermore, the Canny Edge Detector poorly denoises salt noise and salt-and-pepper noise and does not conserve the edges. Thus, Non-Local Mean and Median Blur filters were applied to the images to remove noise while preserving the edges; this is crucial for the feature extraction step.

Polynomial regression—hyperparameter tuning to reach the least RMSE on validation set yielded an optimal polynomial degree of 2 and mean square and RMS errors on the test set of 0.3498‰ and 0.5914‰, respectively.

Decision tree regression—hyperparameter tuning procedure to find values with the least validation set error produced a optimal tree depth of 10 for the least RMSE on validation loss. A mean square error of 0.2609‰ and RMSE of 0.5108‰ were achieved on the test set.

Random forest regression—hyperparameter tuning procedure to find the optimal number of trees and subset (number) of features with the least RMSE on validation was achieved for a optimal number of features for input considered the full set, the five input features; the optimal number of trees was 100 results in least validation loss. This results in a mean square error of 0.2560‰ and an RMSE of 0.5060‰ on the test set.

Support Vector Regression—hyperparameter tuning to find the optimal hyperparameter combination that offers the least RMSE on the validation set is produced for C=50, coef0=5, ϵ=0.3, γ=0.1, kernel = linear, resulting in a mean square error on the test of 0.5925‰ and an RMSE of 0.7698‰.

Fully connected network regression—hyperparameter tuning to find the hyperparameter combination with the least RMSE; on the validation set, the optimal number of hidden layers is 4 and 20 neurons for each hidden layer. A learning rate (η) of 0.003, with Stochastic Gradient Descent as the optimizer, results in a mean square error and RMSE on the test set of 0.4050‰ and 0.6364‰, respectively.

### 5.2. Deep Learning

The deep learning architecture does not required hyperparameter optimization, since predefined architectures are available, e.g., at Google, Facebook, Microsoft, Universities, and at other research and technological institutions.

In the case of GoogLeNet architecture, the mean squared error on the test set is 0.0082‰ and the RMSE is 0.0910‰. For the ResNet34 architecture, a mean squared error of 0.0032‰ and an RMSE of 0.057‰ were reached on the test set, also indicating high confidence level—99.943%.

### 5.3. Benchmarking

**Figure 9:**
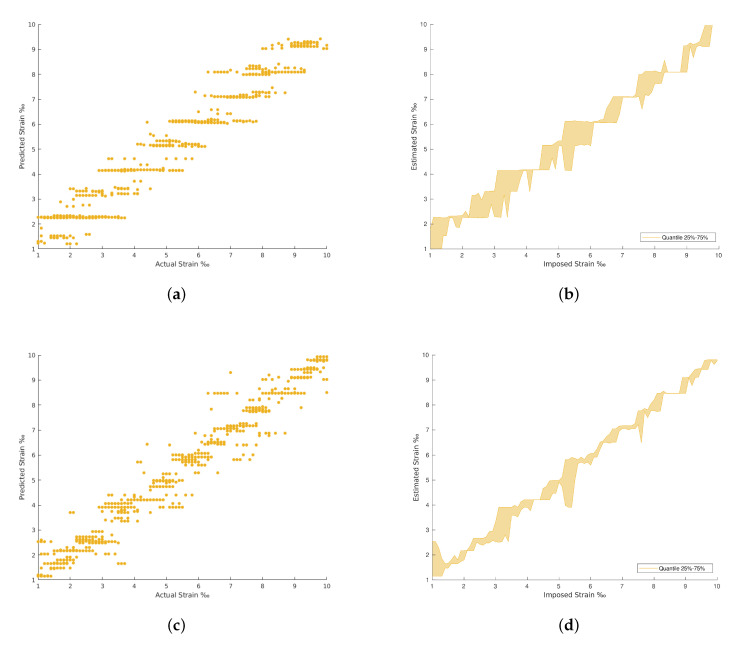
Polynomial Regression and Decision Tree Regression results. (**a**) Polynomial Regression predictions. (**b**) Polynomial Regression predictions by quantile variations. (**c**) Decision Tree Regression predictions. (**d**) Decision Tree Regression predictions by quantile variations.

**Figure 10:**
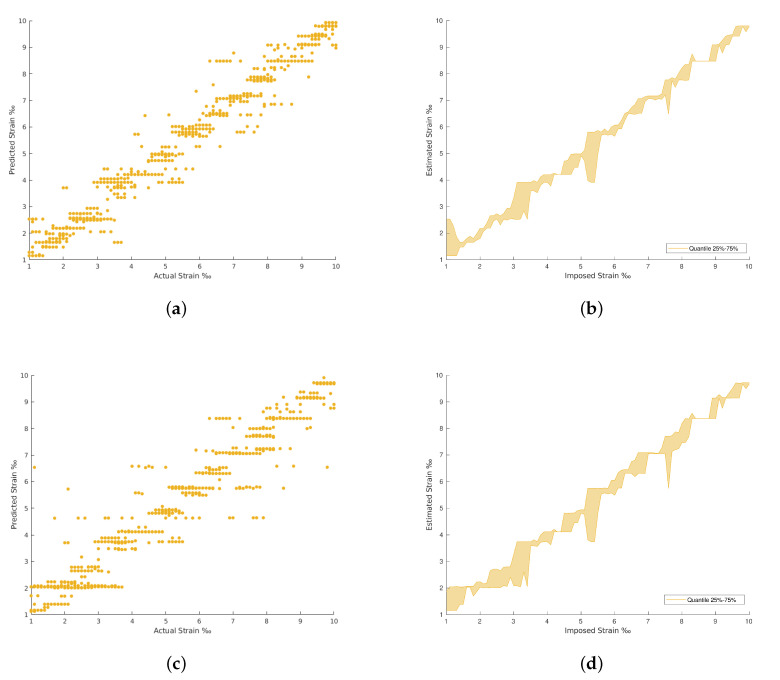
Fully connected neural network (FCNN) and Random Forest results. (**a**) Random Forest Regression predictions. (**b**) Random Forest Regression predictions by quantile variations. (**c**) FCNN with regression predictions. (**d**) FCNN with regression predictions by quantile variations.

**Figure 11:**
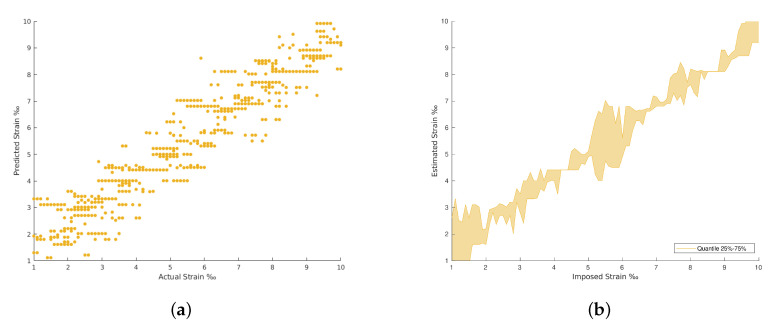
Support Vector Regression (SVR) results. (**a**) Support Vector Regression predictions. (**b**) Support Vector Regression predictions by quantile variations.

**Figure 12:**
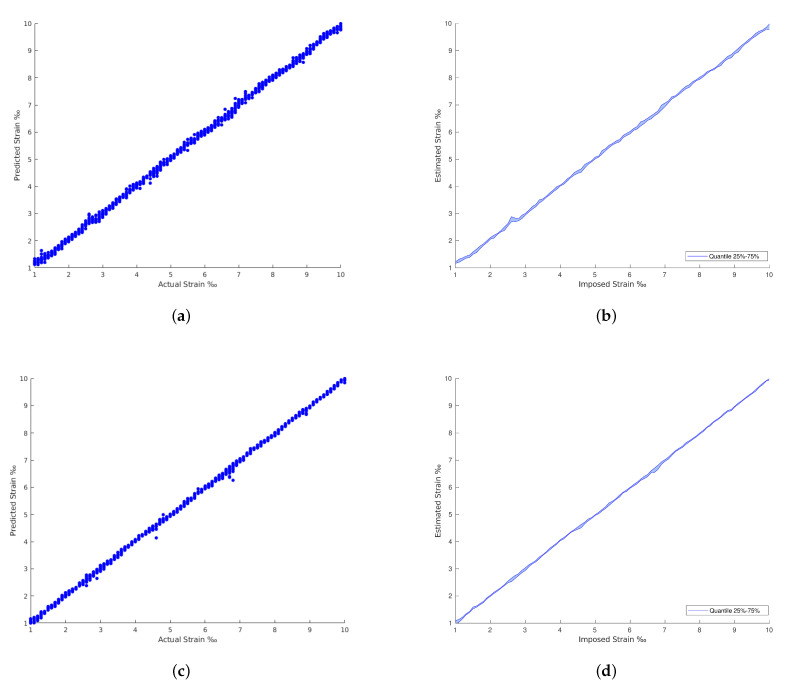
ResNet and GoogLeNet results. (**a**) GoogLeNet with regression predictions. (**b**) GoogLeNet with regression predictions by quantile variations. (**c**) ResNet with regression predictions. (**d**) ResNet with regression predictions by quantile variations.

The results achieved in all the architectures evaluated are provided in Table 2. The confrontation between the imposed and the estimated strain for each strain level are plotted from Figure 9, Figure 10, Figure 11 and Figure 12. The comparison between the imposed and the estimated strain enables the accuracy to be calculated—in this case, computing the mean square and RMS errors. Instead, the precision was calculated by analyzing the dispersion of the results obtained.

In general, the traditional machine learning methods show higher errors for the strain estimation, resulting in lower explained variances between the estimated, [$\hat{y}$], and imposed, [*y*], strain levels. This fact is well identified by the dispersion presented in the Quartiles analysis, between 25% and 75% of results, and plotted in Figure 9, Figure 10, Figure 11 and Figure 12 (b and d). On the other hand, the deep Learning algorithms tested, ResNet and GoogLeNet, had better performance in the validation and test datasets compared to machine learning algorithms for computer vision. ResNet34 managed to outperform all the benchmark algorithms tested, achieving an RMSE of 0.057‰ on the test set. In addition, a high level of confidence was assured, over 99.9%, on new measures, as shown by the lower dispersion of results presented in Figure 12.

**Figure 13:**
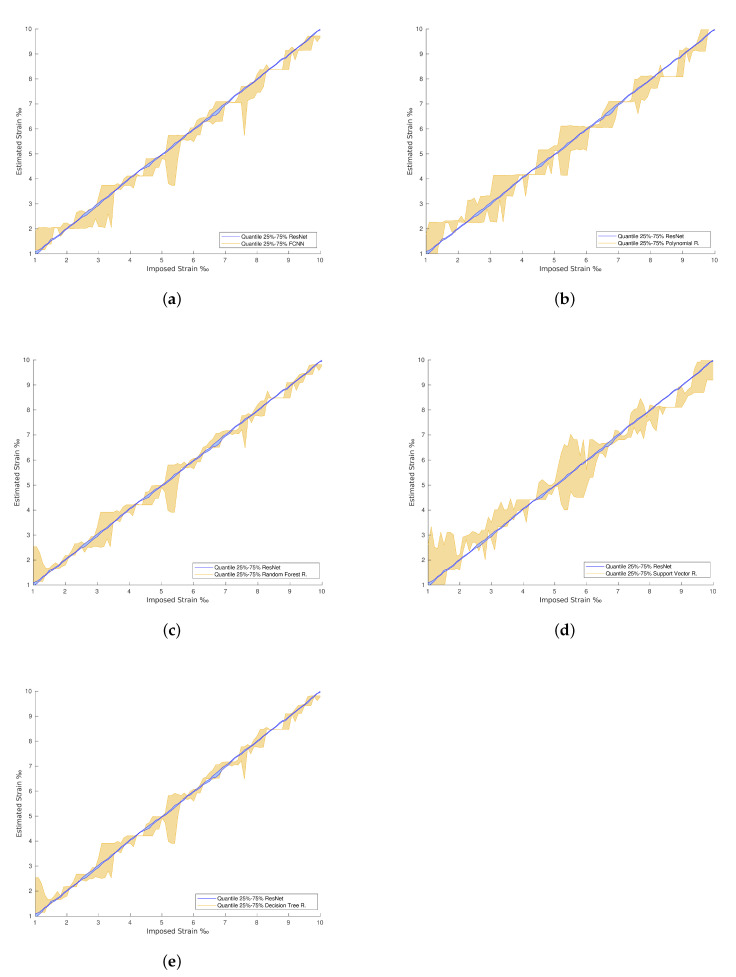
ResNet with regression vs. machine learning predictions. (**a**) ResNet with regression vs. FCNN with regression predictions. (**b**) ResNet with regression vs. Polynomial Regression predictions. (**c**) ResNet with regression vs. Random Forest Regression predictions. (**d**) ResNet with regression vs. Support Vector Regression predictions. (**e**) ResNet with regression vs. Decision Tree Regression predictions.

**Figure 14:**
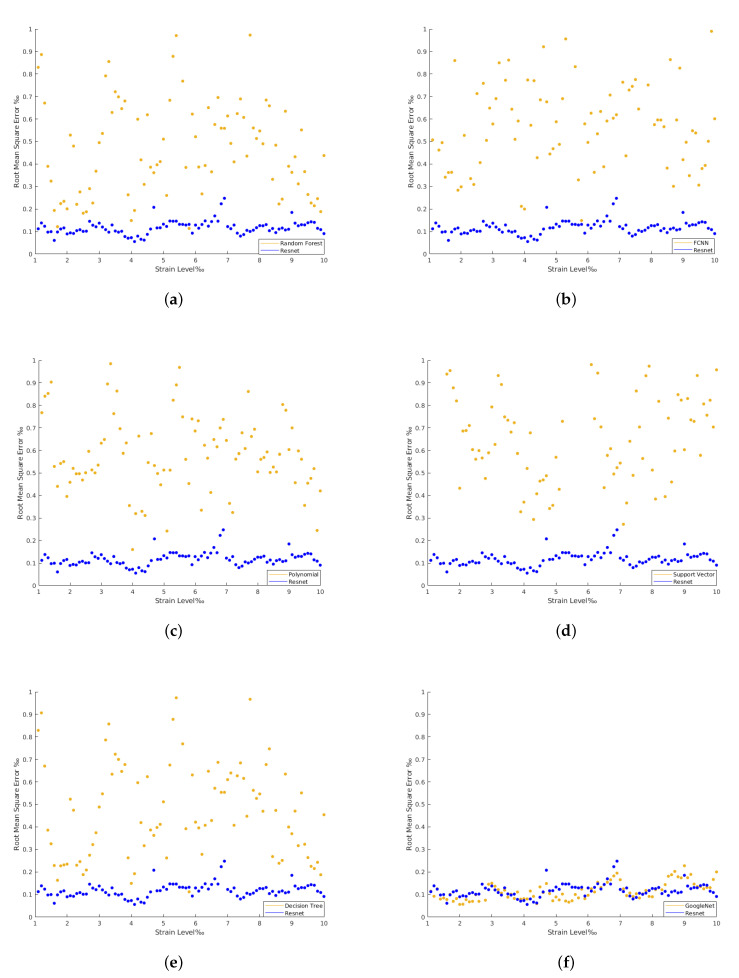
ResNet with regression RMSE vs. the rest. (**a**) ResNet with regression RMSE vs. Random Forest Regression RMSE. (**b**) ResNet with regression RMSE vs. FCNN with regression RMSE. (**c**) ResNet with regression RMSE vs. Polynomial Regression RMSE. (**d**) ResNet with Regression RMSE vs. Support Vector Regression RMSE. (**e**) ResNet with regression RMSE vs. Decision Tree Regression RMSE. (**f**) Resnet with regression RMSE vs. GoogLeNet with regression RMSE.

The confrontation between the dispersion of the best machine learning method *versus* the dispersion of ResNet34 is presented in Figure 13. Once again, it is clear that the machine learning methods estimations have higher dispersion in their prediction when compared to the more stable and robust-to-noise deep learning methods. Further, Figure 14 demonstrates that the RMSE of the deep learning methods remains low for the strain levels evaluated. The time required for the tests when applying machine learning methods on a local machine, with Intel(R) Core(TM) i5-7500T CPU @ 2.70 GHz, is 0.15 s. For deep learning algorithms, the optimal solution required 200 epochs to 800 epochs or more to be run during training. This takes 80 s of training time per epoch on an 8-core Tensor Processing Unit (TPU), around 0.1 s of test time per image, and 2 s on the local machine. For this situation, the RMSE approaches zero along training as the number of epochs increases.

## 6. Conclusions

This paper presents contact-free architecture for measuring strain in CFRP laminate based on computer vision. The architecture was designed and implemented to benchmark algorithms of machine and deep learning. A dataset of images was produced to mimic acquisition with a low-resolution camera, also considering noise filters to mimic acquisition in real scenarios. The use of controlled synthetic images enables the algorithms to be tested without interference from the possible errors associated with the acquisition of real cases. Machine learning and deep learning approaches were benchmarked and the main conclusions are as follows:

- The architecture based on deep learning clearly provides the most feasible and accurate solutions, with high confidence measurements of the strain evolution during the pre-stress application;
- The deep learning algorithms proved to be robust to noise on the images, unlike the machine learning solutions tested, where the noise affects the quality of the results obtained;
- The architecture based on the ResNet34 deep learning algorithm achieved better performance, reaching the lowest root mean square error (RMSE) of 0.057‰ for strain prediction;
- In addition, ResNet34 also reached the highest explained variance, 0.9996, close to the perfect value of 1, and the highest covariance of 7.12 × 10^−6^.

Regarding computation performance, the CPU can be used for inference in deep learning approaches, but the algorithms were trained on the cloud due to huge GPU memory needs and large computational power requirements. In addition, deeper networks, such as ResNet150, i.e., ResNet with 150 layers, were explored. However, its training time per epoch was 1 h on a powerful 8-core Tensor Processing Unit available in Google Cloud, compared to the 580 s per epoch taken by the architecture proposed. It was assumed that deeper networks can have a slightly better performance, but the trade-off will lead to a high computational cost.

## Figures and Tables

**Table 1:** Parameters used for Benchmark.

Algorithm	Number of Layers	Number of Epoch	Learning Rate	Batch Size
GoogLeNet	22	200	0.001	1
ResNet	34	500	0.001	32

**Table 2:** Benchmark results.

Algorithm	Mean Squared Error (‰)	RMS Error (‰)	Explained Variance	Covariance ($\times 10^{-6}$)	R^2^
Polynomial Regression	0.3498	0.5914	0.9493	6.5199	0.9494
Decision Tree Regression	0.2609	0.5108	0.9620	6.6470	0.9622
Random Forest Regression	0.2560	0.5060	0.9631	6.6327	0.9629
Support Vector Regression	0.5925	0.7698	0.9141	6.2753	0.9142
Fully Connected Neural Network	0.4050	0.6364	0.9411	6.7609	0.9392
GoogLeNet + Regression	0.1852	0.4303	0.9989	7.0399	0.9990
ResNet + Regression	**0.0032**	**0.0570**	**0.9996**	**7.1225**	**0.9996**

## Data Availability

Data available on request due to restrictions of privacy or ethical.

## Abbreviations

CFRP	Carbon-Fiber-Reinforced Polymer
RMSE	Root Mean Square Error
